# Rapid Printing of Pseudo-3D Printed SnSe Thermoelectric Generators Utilizing an Inorganic Binder

**DOI:** 10.1021/acsami.3c01209

**Published:** 2023-05-04

**Authors:** Geraint Howells, Shahin Mehraban, James McGettrick, Nicholas Lavery, Matthew J. Carnie, Matthew Burton

**Affiliations:** † Department of Materials Science and Engineering, Faculty of Science and Engineering, Swansea University, Swansea SA1 8EN, United Kingdom; ‡ MACH 1, Faculty of Science and Engineering, Swansea University, Swansea SA1 8EN, United Kingdom; § SPECIFIC-IKC, Department of Materials Science and Engineering, Faculty of Science and Engineering, Swansea University, Swansea SA1 8EN, United Kingdom

**Keywords:** thermoelectrics, tin selenide, SnSe, printing, 3D

## Abstract

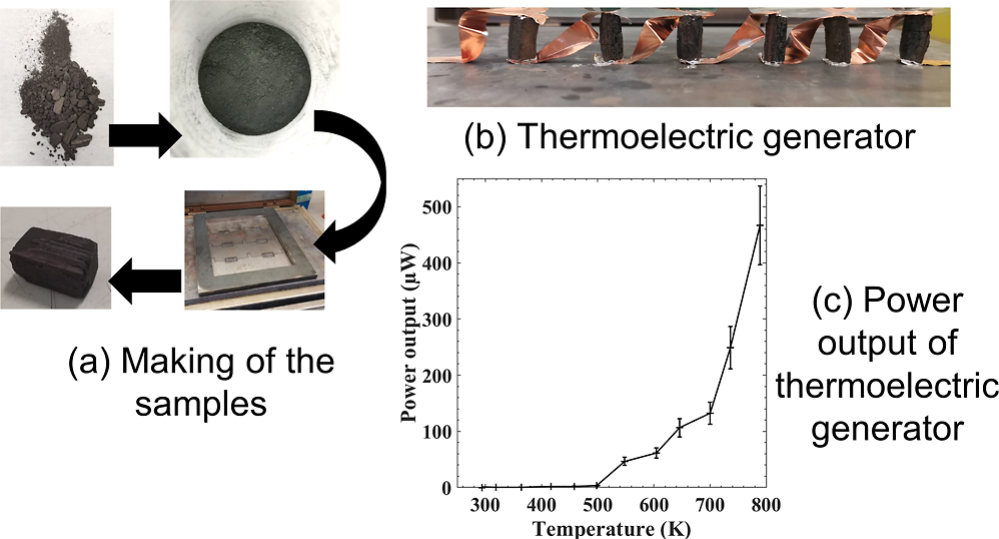

There has been much interest in tin selenide (SnSe) in the thermoelectric community since the discovery of the record zT in the material in 2014. Manufacturing techniques used to produce SnSe are largely energy-intensive (e.g., spark plasma sintering); however, recently, in previous work, SnSe has been shown to be produced via a low embodied energy printing technique, resulting in 3D samples with high zT values (up to 1.7). Due to the additive manufacturing technique, the manufacturing time required was substantial. In this work, 3D samples were printed using the inorganic binder sodium metasilicate and reusable molds. This facilitated a single-step printing process that substantially reduced the manufacturing time. The printed samples were thermally stable through multiple thermal cycles, and a peak zT of 0.751 at 823 K was observed with the optimum binder concentration. A proof-of-concept thermoelectric generator produced the highest power output of any reported printed Se-based TEG to date.

## 1 Introduction

The ever-growing consequences of climate change and the ever-dwindling resource of fossil fuels require a need to transition to sustainable energy solutions. Thermoelectric (TE) materials can play a vital role in lowering fossil fuel usage within the industry.^1^ These materials can be used to recycle waste heat from industry and residential processes into useful electrical energy.^2−4^ TE materials exploit the Seebeck effect, which generates a voltage across a material when a heat differential is applied across the material. This effect happens when there is a movement of charge carriers from the hot side (high-energy state) to the cooler side (low-energy state), holes in p-type, and electrons in n-type, thus generating a difference in potential across the material.^5,6^ TE generators (TEGs) exploit the Seebeck effect in materials to generate power. TEGs consist of alternating p-type and n-type TE legs connected electrically in series and thermally in parallel.^7−9^ The TE performance of a material is characterized by the dimensionless figure of merit (zT), which consists of the Seebeck coefficient (*S*, V K^–1^), electrical conductivity (σ, S m^–1^), thermal conductivity (κ, W m^–1^ K^–1^), and the absolute temperature (*T*, K), as shown in eq 1.^10−12^
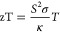
1

Since the discovery of zT ∼ 1 in Bi_2_Te_3_ and PbTe in the 1950s, commercial TEGs have been dominated by bismuth telluride (Bi_2_Te_3_)^13−16^ and its subsequent doped derivatives (Se [n-type] and Sb [p-type]) for low-temperature applications and PbTe for medium temperature applications.^6,17,18^ These materials are good TE resources; however, Te is a relatively earth-rare material with an abundance similar to Pt (1 μg kg^–1^).^19^ If TE technology became extensively used, the tellurium price would become uneconomically viable. The toxicity of Te is also of concern. To alleviate the issues surrounding telluride derivatives, alternative compounds for TE materials need to be used. In 2014, single-crystal SnSe was shown to have a peak zT of 2.6 at 923 K along the *b*-axis; however, the average zT along this axis was only 0.95. Polycrystalline tin selenide (SnSe)^20,21^ with ∼3% atomic Na for Sn replacement has been shown to have a zT as high as 3.1 by Zhou et al. in 2021, making it the highest known zT of any TE material.^22^ While the highest values of zT were reported near the SnSe melting point, the average zT between 400 and 783 K was roughly 2.0, the highest recorded average zT at the time. The high zT comes from the ultralow thermal conductivity of SnSe, thanks to its spring-like structure and its reasonable Seebeck coefficient.^21−37^ The peak zT of SnSe is also at 783 K, making it ideal for use in heavy industry, where waste heat is typically released into the atmosphere.

Current commercial manufacturing techniques for TE materials (e.g., spark plasma sintering and hot pressing) are energy-intensive as they require high temperature and/or high pressure.^25,38^ The manufacturing equipment for these techniques is also extremely expensive. Due to the unfavorable attributes of these techniques, new manufacturing methods need to be explored. One such technique is printing, which can be conducted at room temperature (RT) and pressure, utilizing relatively low-cost equipment and having a high production output. Most TE printing research has focused on screen printing,^11,17,39−42^ inkjet printing,^43−45^ and dispenser printing,^13,46^ which ultimately result in materials of a limited thickness (<1 mm) which are below the thickness required to make efficient TEGs (>5 mm). 3D printing, therefore, needs to be explored for TE materials.^15,47−49^

Pseudo-3D printing of SnSe has already been achieved by Burton et al.;^51^ however, the manufacturing times to make a TE leg were long. There is a need, therefore, to find a new binder for pseudo-3D printing. Sodium silicate is an industrially established binder, being used as an adhesive in the industry for metal sand cast molds and refractory materials,^51^ which is stable up to 1373 K. Here, we investigate the feasibility and performance of using sodium silicate as a binder for SnSe powders to make 3D samples that are pseudo-3D printed in a method akin to casting in a rapid and repeatable manner which does not require additive layers and has a much shorter drying time. For this investigation, the form of sodium silicate used was sodium metasilicate (Na_2_SiO_3_). Na_2_SiO_3_ is a compound which can be dissolved in water, when dried (typically at 383 K), the product produced has a silica portion chemically equivocal to common glass compounds (∼74%).

## 2 Experimental Section

### 2.1 Ball Milling

**Figure 1:**
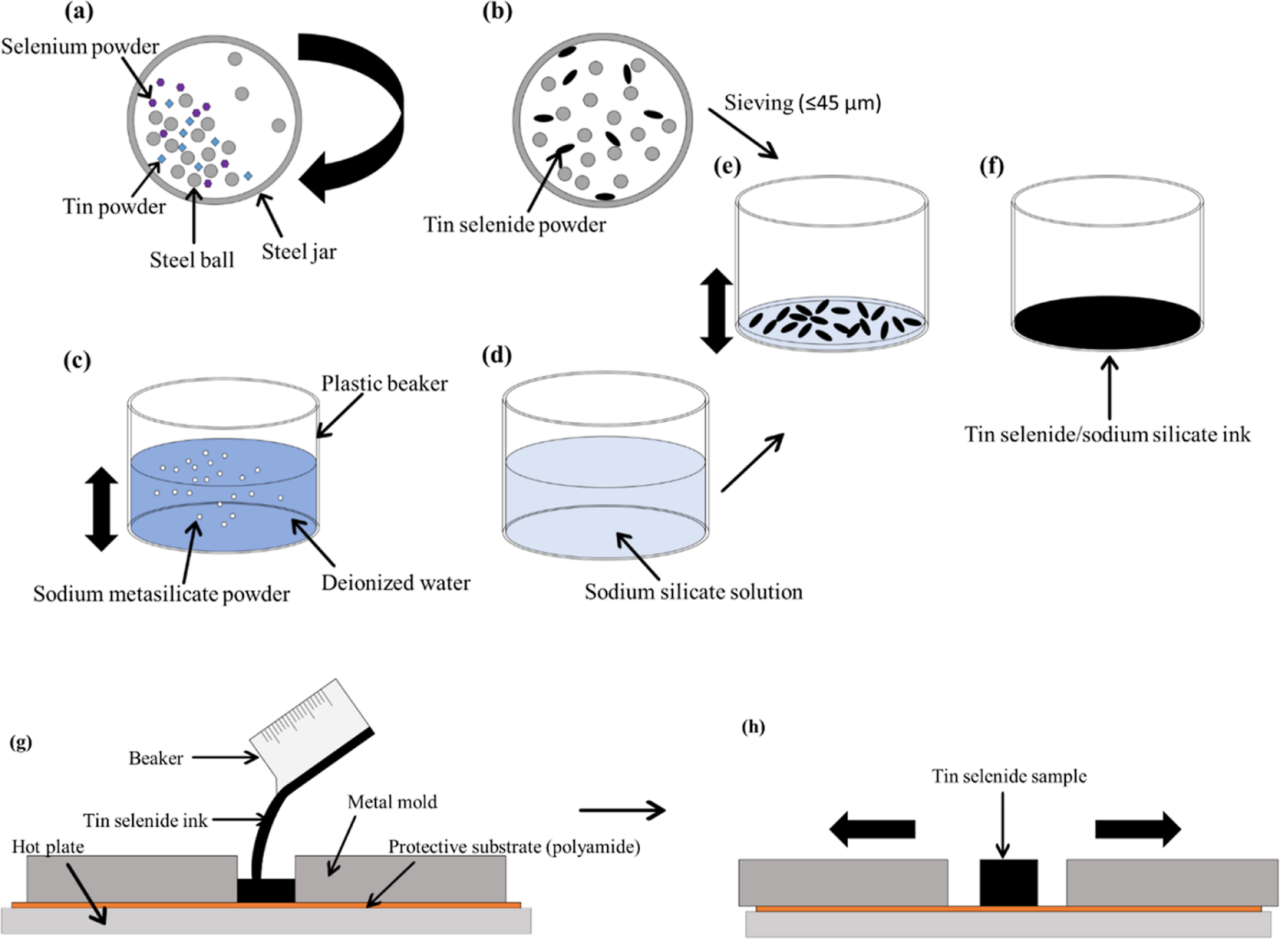
Schematic of the ball milling, ink making, and printing process, with thick black arrows indicating movements: (a) before and (b) after ball milling and (c) making of the sodium silicate solution and the (d) sodium silicate solution. (e) The solution made in (d) is then mixed with the sieved tin selenide powder made in (b) to make the ink that is represented in the last step, (f). (g) Illustration of the molding process for the samples. (h) After molding, the metal molds are removed by gently pulling them apart, revealing the sample.

Mechanical alloying of the raw elements of Sn and Se was achieved via ball milling (Figure 1a,b). Sn (≥99%, Sigma-Aldrich) and Se (≥99.5%, Sigma-Aldrich) were added in equal molar quantities into a stainless-steel jar with 30 stainless-steel ball bearings (10 mm diameter). Another jar was loaded with the same contents to allow the mill to be balanced. The jars were then secured in a planetary ball mill (PULVERISETTE 5/2), and a spin speed and time of 200 rpm and 30 min were used, respectively, followed by a 30 min rest period. This was repeated 60 times, giving a total ball milling time of 60 h. After each spin cycle, the spin direction was also reversed. The ball-milled powder was afterward sieved in a 45 μm Endecotts sieve to ensure that all SnSe particles were 45 μm or below. A SEM image of this powder can be seen in Figure S4d, revealing a variety of <45 μm particles of various shapes.

### 2.2 Pseudo-3D Printing

Binder solutions were made by mixing Na_2_SiO_3_ crystalline powder (Sigma-Aldrich) with deionized water to make 1.5, 2, 3, 4, and 5% by weight mixtures. Solutions were mixed with a vortex genie agitator until full dissolution was achieved (Figure 1c,d). For each binder concentration, ∼2.83 g of the binder solution was then added to ∼6.05 g of ball-milled SnSe (binder solution to SnSe powder weight ratio of ∼1:2.14) and agitated on a vortex genie until a uniform ink was formed (Figure 1e), which involved no change in liquid concentration. Silicon release spray was sprayed into a metal mold which was placed onto a hot plate set at 353 K. Inks were then poured into the metal mold and left for <2 h to dry and form cuboids that measured ∼1 cm × 1 cm × 2 cm (Figure 1g). After drying, the mold was pulled apart, resulting in cuboid samples (Figure 1h). The samples were then cured in an Ar tube furnace for 1 h at 873 K with an Ar flow rate of 1 L min^–1^, after which the furnace heating coil was turned off, allowing the samples to cool to RT. For X-ray diffraction (XRD) analysis, a pure Na_2_SiO_3_ sample was also made using the same method. Here, 7 g of Na_2_SiO_3_ powder was mixed with 14 g of water.

### 2.3 Materials Characterization

XRD was performed on a Bruker D8 diffractor with Cu Kα radiation. Scanning electron microscopy (SEM) and energy-dispersive X-ray spectroscopy (EDX) were performed on a JOEL 7800F field emission gun scanning electron microscope with an Oxford Laboratory EDX attachment. X-ray photoelectron spectroscopy (XPS) was performed on a Kratos Axis Supra instrument, and data were processed in CasaXPS (2.3.24PR1.0). Samples were mounted in electrical contact with the stage. XPS was sampled to a depth of <10 nm using a monochromatic Kα source (225 W, 15 mA) with a footprint of 300 × 700 μm and a pass energy of 40 eV, with the GL(30) line shape.

### 2.4 Thermoelectric Characterization

Electrical conductivity and Seebeck coefficients were measured in a He atmosphere using a ULVAC ZEM3. The uncertainty of electrical conductivity was ±3% and that of the Seebeck coefficient was ±4%.^52^ Thermal diffusivities (*D*) were determined using a Netzsch 457 laser flash analyzer with Al_2_TiO_5_ sample holders with SiC caps for solid samples, Ø 11 mm × 1.5 mm, and using the Cowon + pulse correction diffusivity model. This was calibrated with a 10 mm Ø Pyroceram 9606 calibration standard. The uncertainty of thermal diffusivity was ±3%.^52^ Densities were determined using the method of hydrostatic weighing that uses the Archimedes principle, with results reported in Table S1. Sample dimensions were measured before and after measurement, with no observable change. The uncertainty in density measurements was ±1%.^52^

## 3 Results and Discussion

### 3.1 Materials Characterization

**Figure 2:**
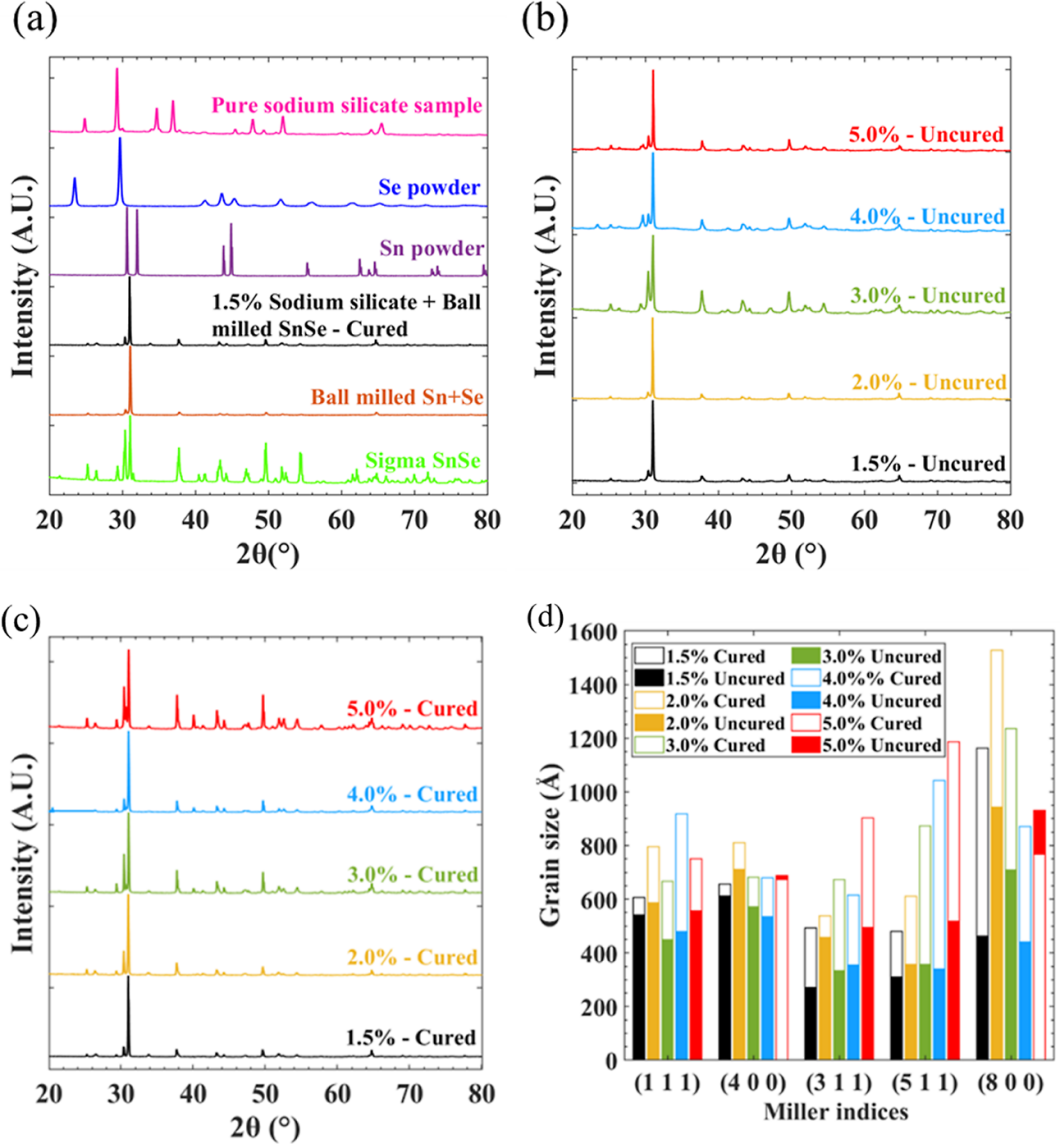
XRD: (a) constituent materials, ball-milled SnSe and commercially available SnSe, (b) uncured printed samples, (c) cured printed samples, and (d) grain growth of samples; full bars represent samples before curing, while line bars (empty bars) represent samples after curing; the corresponding peaks to the miller indices in (d) are given in the Supporting Information (Figure S1c).

To prove that ball milling was sufficient to produce SnSe from Sn and Se powders, XRD was conducted (Figure 2a). The ball-milled SnSe lacks the peaks present in the initial Sn and Se powders, indicating that there are negligible to no unreacted elemental powders. Peaks from the ball-milled SnSe powder spectrum align to peaks in the commercially bought SnSe (Sigma-Aldrich), albeit with a degree of orientation. These can be assigned to the low-temperature orthorhombic phase of SnSe which belongs to the *Pnma* space group. Lattice constants for the ball-milled SnSe were derived to be 11.53, 4.17, and 4.46 Å for the *a*, *b*, and *c* parameters, respectively, which is in line with literature values (crystallographic open database [COD] 1538896, 1537675). The dominant peak at ∼32° corresponds to the (1 1 1) plane, indicating preferred orientation along that plane^53^ (COD 1537675). This could be explained by nonspherical particles, which upon compaction for XRD preferentially orientated. XRD on a pure Na_2_SiO_3_ sample (Figure S1) shows that the peaks present in this pure binder sample are also present when the binder is combined with SnSe; this demonstrates that the binder has not chemically reacted with SnSe.

**Figure 3:**
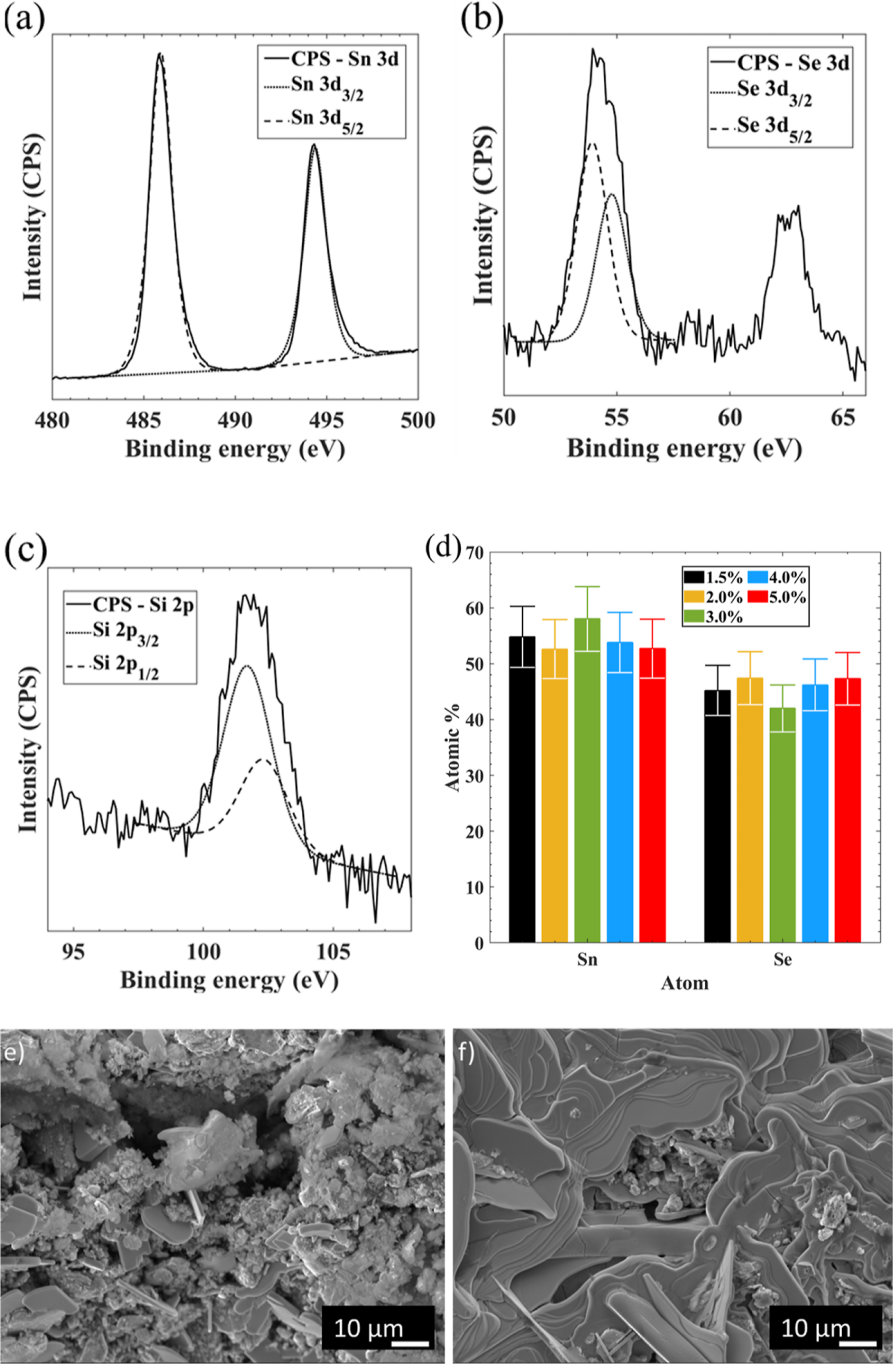
XPS for surface chemistry analysis of printed cured SnSe (1.5% binder) in the regions of (a) Sn, (b) Se, and (c) Si (equivalent uncured spectra can be seen in Figure S2), (d) EDX analysis of printed cured SnSe samples (error bars deduced from error observed from a commercially sourced SnSe), and SEM of printed cured SnSe samples with (e) 1.5% binder and (f) 5% binder (other binder percentages can be seen in Figure S4).

XRD spectrum for samples precure (Figure 2b) and postcure (Figure 2c) show that after curing in Ar, the peaks become taller and narrower, indicating an increase in grain size. Although at higher percentages, the aforementioned (1 1 1) plane SnSe peak is still prevalent, it is not as dominating, indicating that at higher concentrations, Na_2_SiO_3_ may hinder SnSe particle orientation. Figure 2d shows the grain growth of samples, precure to postcure. The grain size was determined using the Scherrer equation assuming spherical particles. The grain size in general increased when cured; however, there were instances where the grain size marginally decreased with 5% Na_2_SiO_3_. All grain sizes, however, were significantly smaller than the larger <45 μm particles seen in SEM images (Figures 3 and S4), indicating subparticle grains. This happened for the (4 0 0) and (8 0 0) planes, implying that at higher binder concentrations, SnSe grain growth may be hindered. Figure S1 shows the XRD spectra for the pure Na_2_SiO_3_ sample compared to the 1.5% Na_2_SiO_3_ formulation, 5.0% Na_2_SiO_3_ formulation, and ball-milled Sn and Se, with no binder. They show that the peaks for the pure Na_2_SiO_3_ sample become more prevalent in the higher binder 5.0% formulation, including that near the SnSe (8 0 0) peak. This may have artificially broadened the SnSe peak, resulting in an artificially reduced grain size calculation. The 1.5% to pure Na_2_SiO_3_ comparison spectra showed that the main dominant peak of Na_2_SiO_3_ at ∼29.3° was also much less prevalent compared to the SnSe (4 0 0) peak at 31.08°.

XPS showed that the surface of the SnSe samples was heavily oxidized. The spectrum of cured Sn (Figure 3a) shows that there is a peak at 485.83 eV, which is in line with the value of 486 eV that is associated with SnSe.^54^ SnO_2_, however, is also within the same region.^55^ The same peak also occurs in the uncured spectrum (Figure S2a). There is also a peak at 494.34 eV, which is associated with Sn oxides of SnO_2_ and SnO,^55^ which can also be seen in the uncured spectrum. There is a peak at 53.9 eV for cured Se (Figure 3b), which is closely associated with the peak of SnSe from previous literature.^56^ This peak again occurred in the uncured spectrum (Figure S2b). The peaks for Si, cured and uncured (Figures 3c and S2c, respectively), imply that Si is in an oxide form, from Na_2_SiO_3_. XPS data shows that at the surface, the SnSe chemistry is greatly Sn-rich.

EDX was used to confirm the bulk elemental composition of the sample’s postcure. Much like the XPS results, Figure 3d shows that the samples exhibit a Sn excess throughout the whole binder concentration range, with no obvious trend with binder concentration. When the samples were left in air postcuring, a pale-pink color typical of SeO_2_ appeared in some areas. The glass air trap at the end of the tube furnace collected a pink deposit during curing (EDX of this deposit confirmed only Se, C, and O), implying that some SeO_2_ escaped during curing. The samples also presented the sour, pungent smell characteristic of SeO_2_ that had not been observed from the pure ball-milled powder or starting materials. These observations imply that SeO_2_ is produced due to a reaction between SnSe and Na_2_SiO_3_, leaving samples with a slight Sn excess. EDX analysis of both a commercially sourced SnSe powder (Sigma-Aldrich, 99.995%) and the raw ball-milled powder (Figure S3) indicate a 60:40 atomic % weighting toward Sn over Se. This indicates that the reason for our observed Sn excess could be an issue with an error in our spectrometer.

SEM images can be seen in Figures 3e,f and S4. As the concentration of Na_2_SiO_3_ increases, the amount of Na_2_SiO_3_ flake-like growth also increases. Figure 3e shows how the 1.5% concentration sample has a porous texture. Figure 3f demonstrates how this porosity substantially reduces with the SnSe particles surrounded by Na_2_SiO_3_ with a 5.0% concentration, a potential reason for grain growth hindrance at this binder concentration calculated using the Scherrer equation.

### 3.2 Thermoelectric Characterization

**Figure 4:**
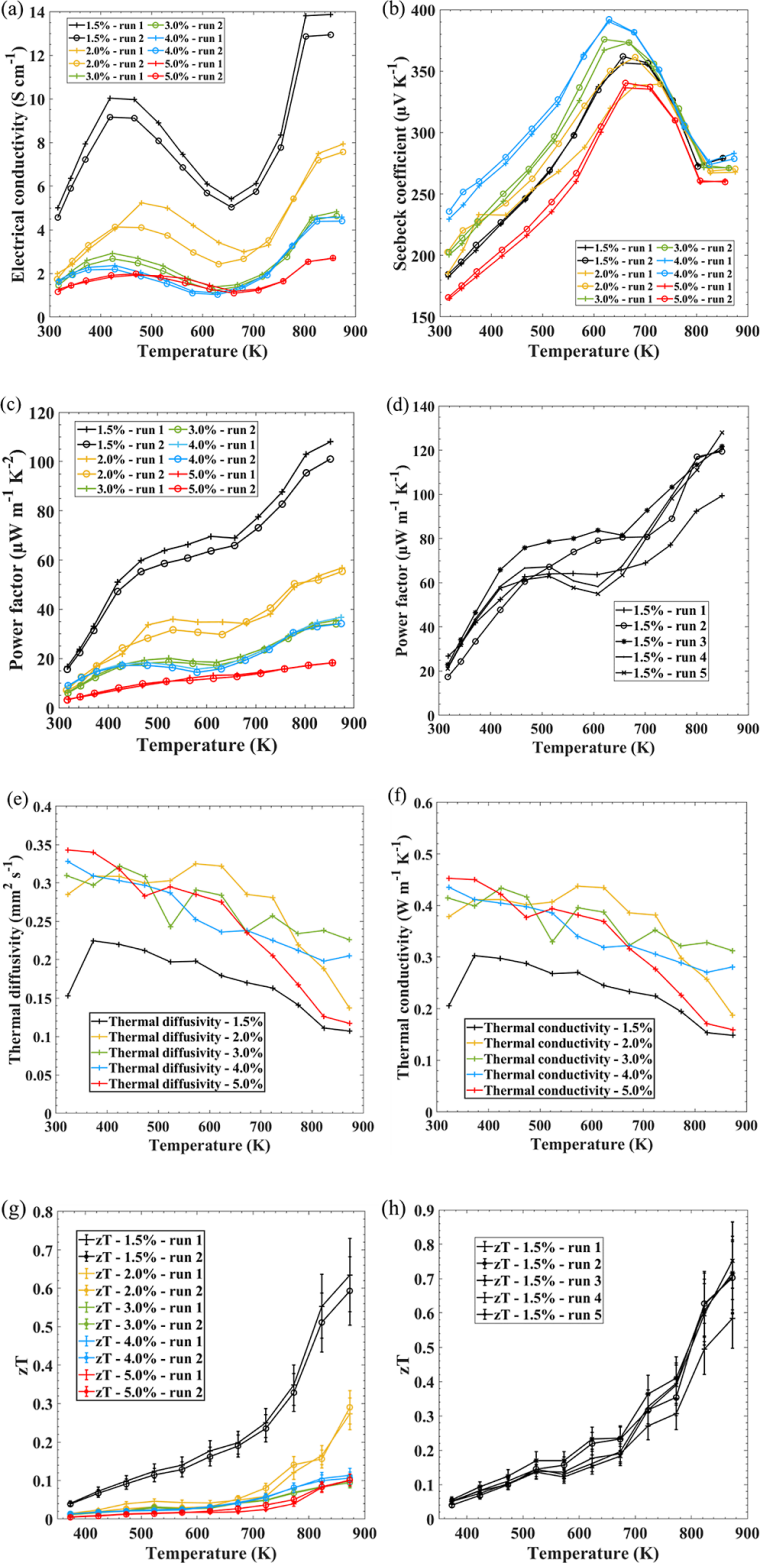
TE performance of pseudo-3D printed SnSe using a Na_2_SiO_3_ binder (cross marks represent the first runs, while the circular marks represent the second runs): (a) electrical conductivity, (b) Seebeck coefficient, (c) power factor, (e) thermal diffusivity, (f) thermal conductivity, (g) zT, with a measurement uncertainty of 15% represented with error bars,^52^ and (h) zT. TE performance over several thermal cycles of pseudo-3D printed SnSe with the 1.5% Na_2_SiO_3_ binder.

Figure 4a shows the electrical conductivity of the printed SnSe samples. All printed samples show a similar trend of electrical conductivity to single-crystal SnSe^23^ above ∼450 K and previously printed work conducted throughout all temperatures.^50,57^ The electrical conductivity reaches a first peak at ∼450 K, subsequently declining until ∼650 K, after which the electrical conductivity increases. The sudden increase in electrical conductivity seen between 750 and 873 K can be explained by the reversible phase change (*Pnma* to *Cmcm*) that occurs in SnSe at around 750 K. The electrical conductivity, however, does appear to be greatly affected by the concentration of Na_2_SiO_3_, with the conductivity decreasing as the amount of binder increases. The sample containing 1.5% Na_2_SiO_3_ is the most conductive sample, hitting a peak conductivity of 13.9 S cm^–1^ at ∼850 K on the first thermal cycle. The second thermal cycle also yielded a similar peak conductivity of 12.9 S cm^–1^ at ∼850 K. This trend in electrical conductivity relative to binder concentration can be explained by observations from the SEM images, where the SnSe particles at higher concentrations are surrounded by the insulating Na_2_SiO_3_, thus removing carrier pathways within the samples, which would otherwise be present at lower concentrations. All samples show typical Seebeck coefficient temperature trends (Figure 4b) demonstrated in previous literature surrounding SnSe.^22,50^ The Seebeck effect hits a peak in each sample at ∼650 K, before dropping. The drop is then halted at around 800 K again due to the phase change that occurs within SnSe. The peak Seebeck coefficient, 392 μV K^–1^ at 600 K, was achieved at a 4% binder concentration. The binder concentration had no visible effect on the trend of the Seebeck coefficient within the samples. Figure 4c displays the power factors (*S*^2^σ). Due to the increased electrical conductivity seen in 1.5% Na_2_SiO_3_ SnSe and the lack of the binder effect on the Seebeck coefficients, the 1.5% binder is seen to have the largest power factor of 108 μW m^–1^ K^–2^ on the first thermal cycle and 101 μW m^–1^ K^–2^ on the second thermal cycle, both at ∼850 K. The power factor relative to the binder concentration also showed a similar trend to electrical conductivity, with the power factor decreasing as the amount of binder increased.

As 1.5% Na_2_SiO_3_ SnSe exhibited the highest power factor, the reproducibility and thermal hysteresis were tested with that concentration of the binder with a new sample. Five thermal cycles from RT to 850 K were conducted. The power factor (Figure 4d) increased to 127.9 μW m^–1^ K^–2^ at ∼850 K after five thermal cycles; this is likely due to the continued grain growth of the SnSe through thermal cycles, allowing for greater carrier pathways to be formed. The electrical conductivity and Seebeck coefficient of the sample are shown in Figure S5.

The density of the printed samples (Table S1) reveals a stable density over the binder concentrations studied. The average density of 5.3 g cm^–3^ is 85.8% of the theoretical density of SnSe (6.18 g cm^–3^), with a porosity of 14.2%. In practice, the theoretical density is not achieved and single-crystal SnSe produced via Bridgman crystal growth was shown to only have a density of 5.43 g cm^–3^.^23^ Compared to these SnSe Bridgman growth single crystals, the printed samples have a density of ∼97.6% and a porosity of 2.4%.

The thermal conductivity (Figure 4f) of the printed SnSe samples was determined through the product of thermal diffusivity (Figure 4e), density (Table S1), and specific heat capacity obtained from the literature^23^ (κ = *D*_ρ_*C*_*p*_). All values observed are in the range of ∼0.45 to ∼0.15 W m^–1^ K^–1^, with an inverse correlation with the measurement temperature. These numbers are in line with those seen for the *a*-axis of single-crystal SnSe and lower than those seen for the *b*-axis and *c*-axis.^23^ While lower than most thermal conductivity values reported for SnSe in the literature (Figure S6a), most likely due to higher porosity, they are similar to many reported values. Electronic thermal conductivity (κ_e_) values were determined using the Wiedemann–Franz law (κ_e_ = *L*·σ·*T*, with *L* = 1.5 × 10^–8^ V^2^ K^–2^), and the lattice thermal conductivity (κ_L_) was determined by κ_L_ = κ – κ_e_,^23,58^ with the values reported in Figure S7. The 5.0 and 1.5% formulations produced the highest and lowest thermal conductivity values, respectively; however, there is no obvious trend between the binder concentration and thermal conductivity. A peak zT (Figure 4g) of ∼0.65 at 873 K was achieved in the 1.5% binder sample. zT over several thermal samples for the 1.5% binder (Figure 4h) shows an initial increase in zT after one thermal cycle. zT is seen to be stable over the following thermal cycles, with a peak of 0.75 at 823 K observed. While a zT of 0.75 is significantly lower than the highest zT ever reported for SnSe (3.1) due to lower electrical conductivity and Seebeck coefficient values,^22^ this value is comparable with many other literature-reported values for polycrystalline SnSe (Figure S6b).

### 3.3 Thermoelectric Generator

**Figure 5:**
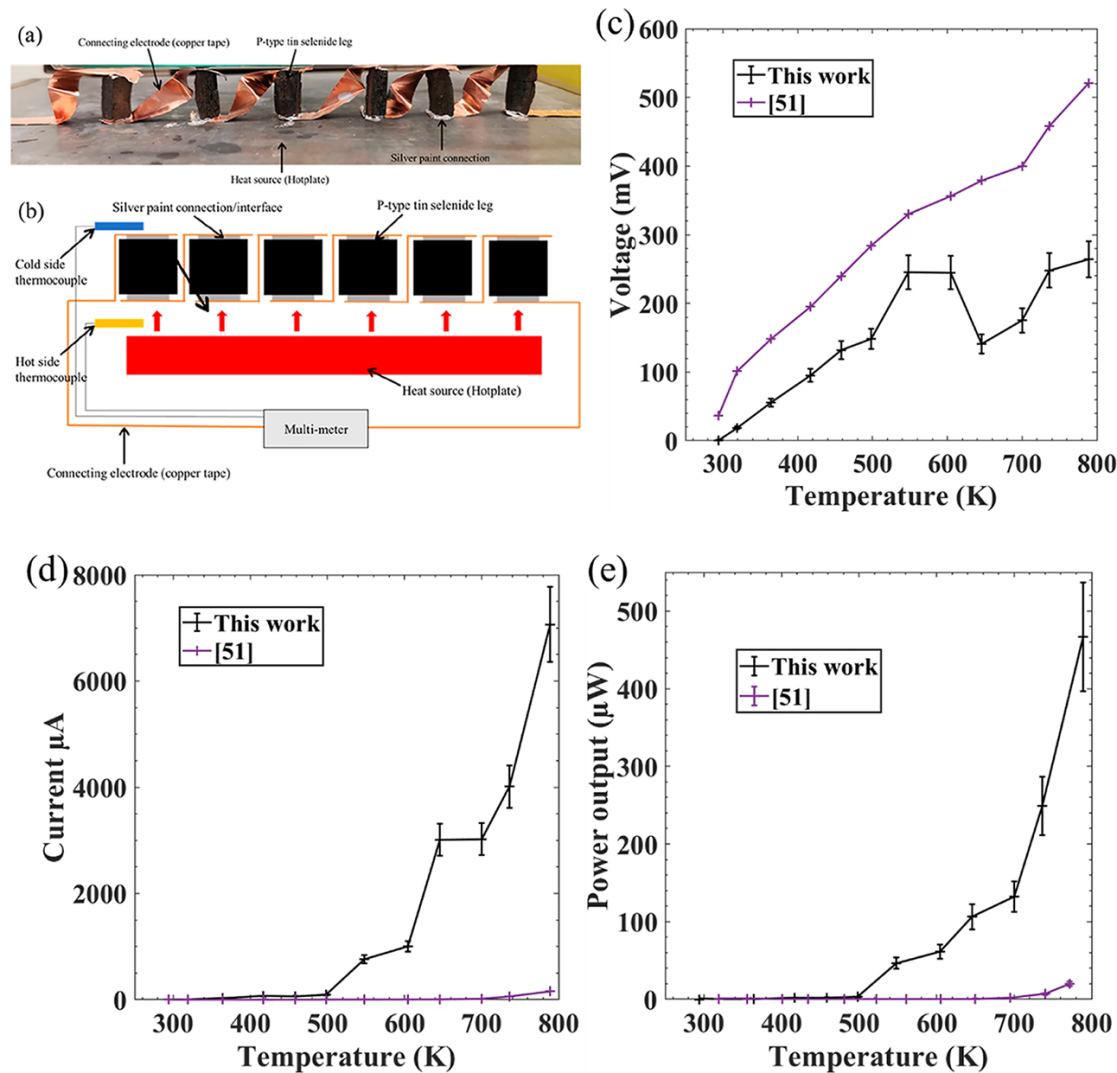
(a) Photo of the SnSe TEG, (b) schematic of the TEG characterization setup, (c) open-circuit voltage (*V*_OC_) output of TEG, (d) short-circuit current (*I*_SC_) output of the TEG, and (e) peak power outputs of the TEG (assuming maximum power = *I*_SC_*V*_OC_/4).^60^

A proof-of-concept p-type-only TEG was also created. This involved pseudo-3D printing six SnSe p-type legs with 1.5% Na_2_SiO_3_. The six p-type legs were then connected in a *Z*-type connection approach as seen in Figure 5a, with a schematic of the testing environment shown in Figure 5b. A hot plate was used as a heat source, thermocouples were used to measure both the hot- and cold-side temperatures, and a multimeter was used to determine the short-circuit current (*I*_SC_) and open-circuit voltage (*V*_OC_). Peak power outputs of the device were calculated assuming maximum power = *I*_SC_*V*_OC_/4. Copper tape was used as the connecting electrode with silver paint to minimize parasitic contact resistance, a problem encountered with TE materials.^59^ The results can be seen in Figure 5 and Table S2. The voltage produced by the device (Figure 5c) increases rapidly from RT before hitting a first peak of 245 mV at 548 K. The voltage then decreases before hitting a new, higher peak of 264 mV at 789 K. This pattern in voltage is the same pattern seen with the Seebeck coefficients, with the brief drop in voltage as a result of the phase change from *Pnma* to *Cmcm*. Current (Figure 5d) increases rapidly from 498 K onward, reaching a peak of 7065 μA at 789 K. Figure 5e compares the power output from this device to a previous printed SnSe publication.^50^ A substantial improvement in peak power output is seen from 20 to 467 μW. The improvement in performance is most likely due to an improvement in TEG design, through the use of silver paint to reduce the contact resistance between the printed SnSe legs and the Cu contacts. This can be seen through the substantial increase in I_SC_ of the device as seen in Figure 5d.

## 4 Conclusions

SnSe ink was created using an aqueous solution with varying concentrations of Na_2_SiO_3_, to which SnSe powder was added. A rapid pseudo-3D printing technique was used to produce samples up to 1 cm × 1 × cm × 2 cm, where ink was poured into a reusable mold with no need for additive manufacturing layers. This technique proved to be repeatable, reproducible, and rapid (any size from a single poor into a mold and then left to cure for <2 h). The 1.5% binder concentration sample resulted in the highest power factor of 127.9 μW m^–1^ K^–2^ at 850 K, which was observed on the fifth thermal cycle. A peak zT of 0.75 at 823 K was observed in the same sample. The samples also showed no drop in performance after having undergone multiple testing cycles, implying that Na_2_SiO_3_ can securely hold the SnSe together, even at lower concentrations. A proof-of-concept p-type TEG was also produced, producing a peak power output of 467 μW at 789 K, a substantial improvement on the previous 3D printed SnSe TEG^50^ and, to date, the highest reported power output of any printed Se-based TEG.^48^

## Supporting Information Available

XRD, XPS, and EDX spectra; SEM images; performance results; and TEG characterization (PDF)

## Abbreviations

XRD: X-ray diffraction
EDX: energy-dispersive X-ray spectroscopy
XPS: X-ray spectroscopy
