# Supplementary material for: Rapid Printing of Pseudo-3D Printed SnSe Thermoelectric Generators Utilizing an Inorganic Binder
Source: ACS Appl Mater Interfaces. 2023 May 4;15(19):23068–76. doi: 10.1021/acsami.3c01209 (PMC10197076; doi:10.1021/acsami.3c01209)
Supplement: Supplementary file 1 — am3c01209_si_001.pdf [file am3c01209_si_001.pdf]

Supporting Information - Rapid Printing of Pseudo-3D Printed SnSe Thermoelectric Generators Utilising an Inorganic Binder

Geraint Howells[1], Shahin Mehraban[2], James McGettrick[3], Nicholas Lavery[2], Matthew J. Carnie[3], Matthew Burton[3]

[1]Department of Materials Science & Engineering, Faculty of Science and Engineering, Swansea University, Swansea University, Fabian Way, Swansea SA1 8EN, UK

[2]Materials Advanced Characterization Centre, Faculty of Science and Engineering, Swansea University, Fabian Way, Swansea SA1 8EN, UK

[3]SPECIFIC-IKC, Department of Materials Science & Engineering, Swansea University, Faculty of Science and Engineering, Swansea University, Fabian Way, Swansea SA1 8EN, UK

*Corresponding author: m.r.burton@swansea.ac.uk

KEYWORDS

Thermoelectrics, tin selenide, SnSe, printing, 3D

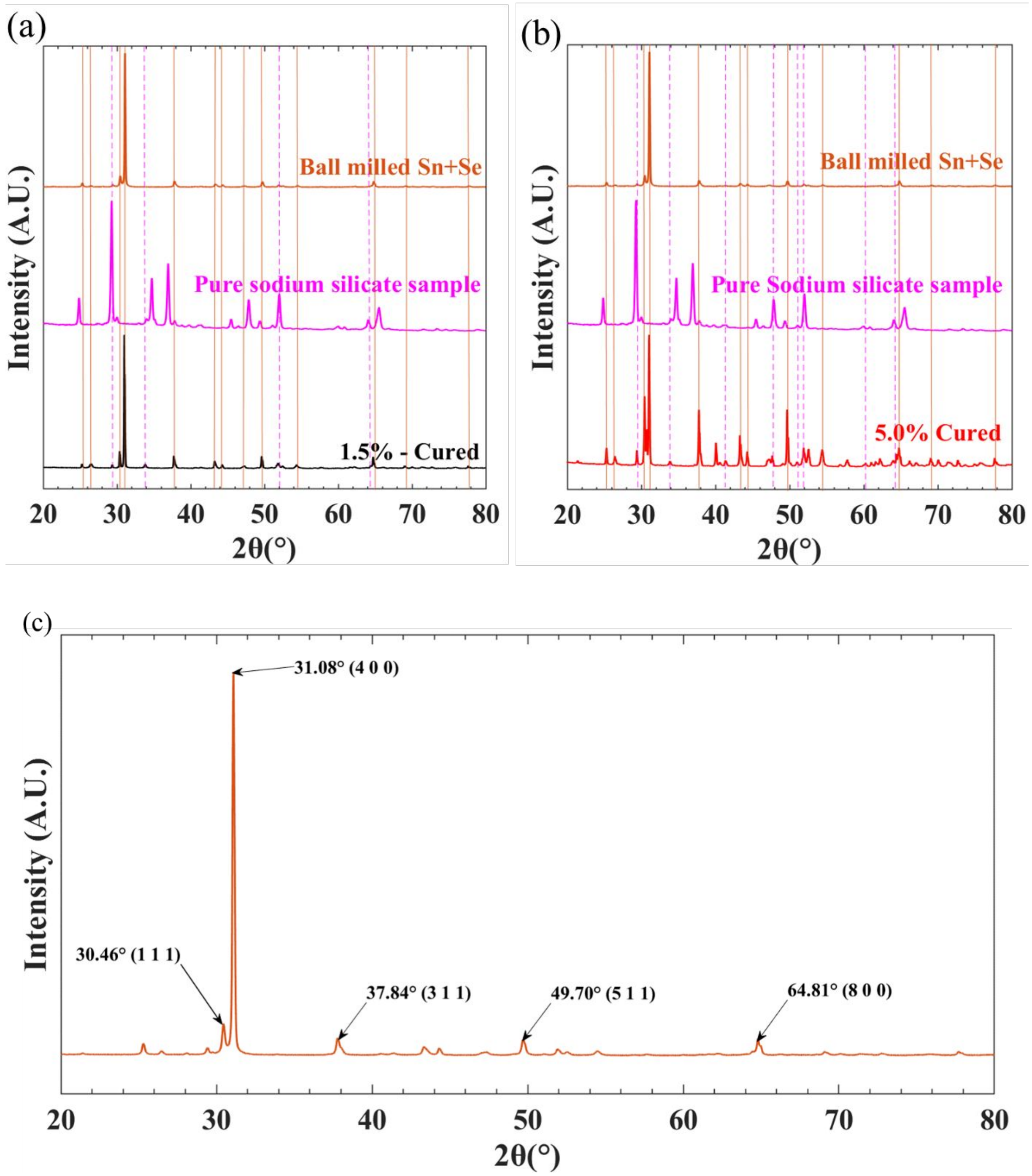

Figure S1. XRD: a) XRD spectra of 1.5%, ball milled Sn and Se, and pure Binder, b) XRD spectra of 5.0%, ball milled Sn and Se, and pure binder, c) XRD spectra of ball milled Sn and Se, with the peaks used for calculating grain growth labelled and corresponding miller indices given (COD 1537675, 1538896)

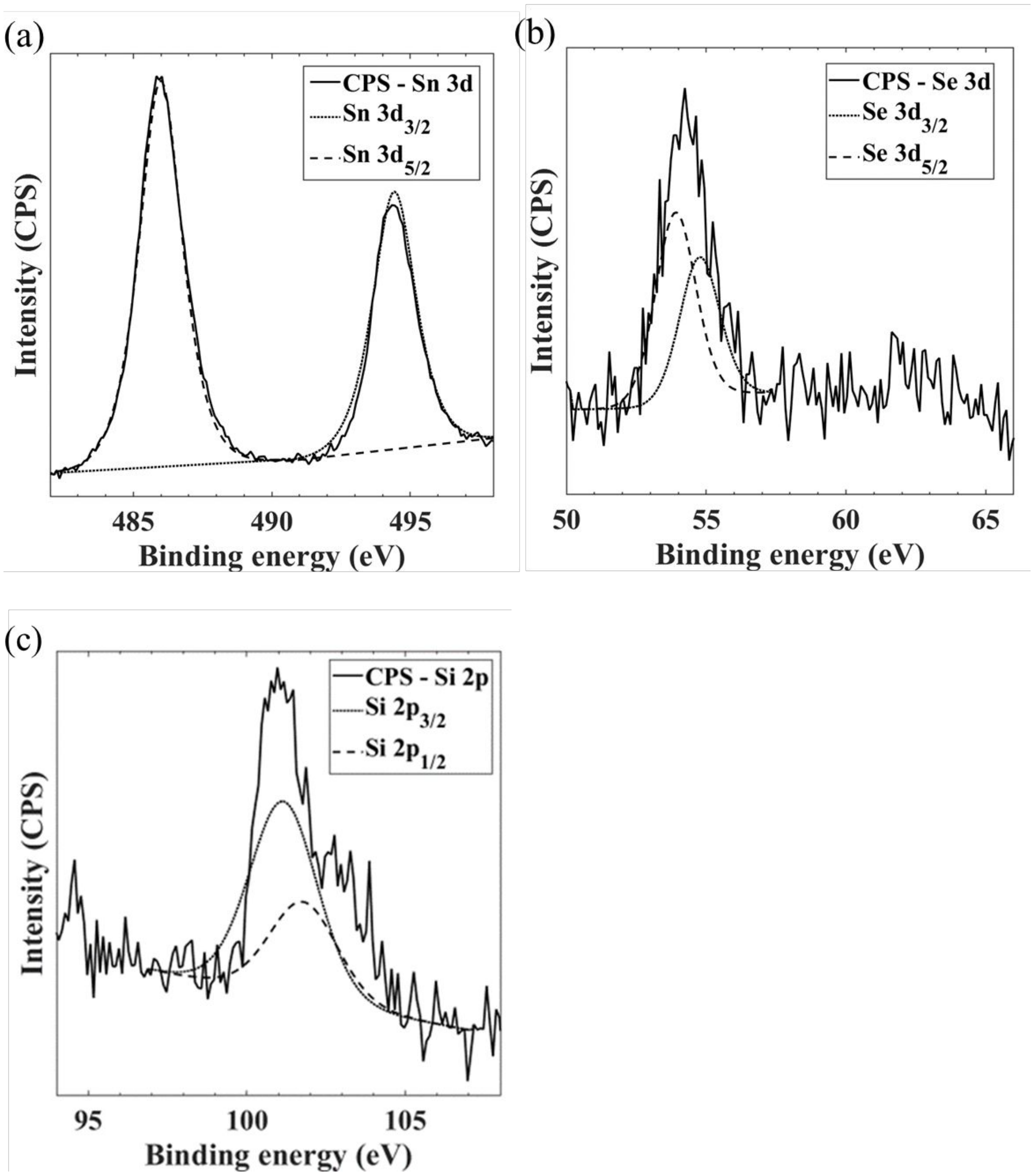

Figure S2: X-ray photoelectron spectroscopy (XPS) for surface chemistry analysis of printed uncured SnSe (1.5% binder) in the regions of a) Sn b) Se and c) Si (equivalent cured spectra can be seen in Figure 2).

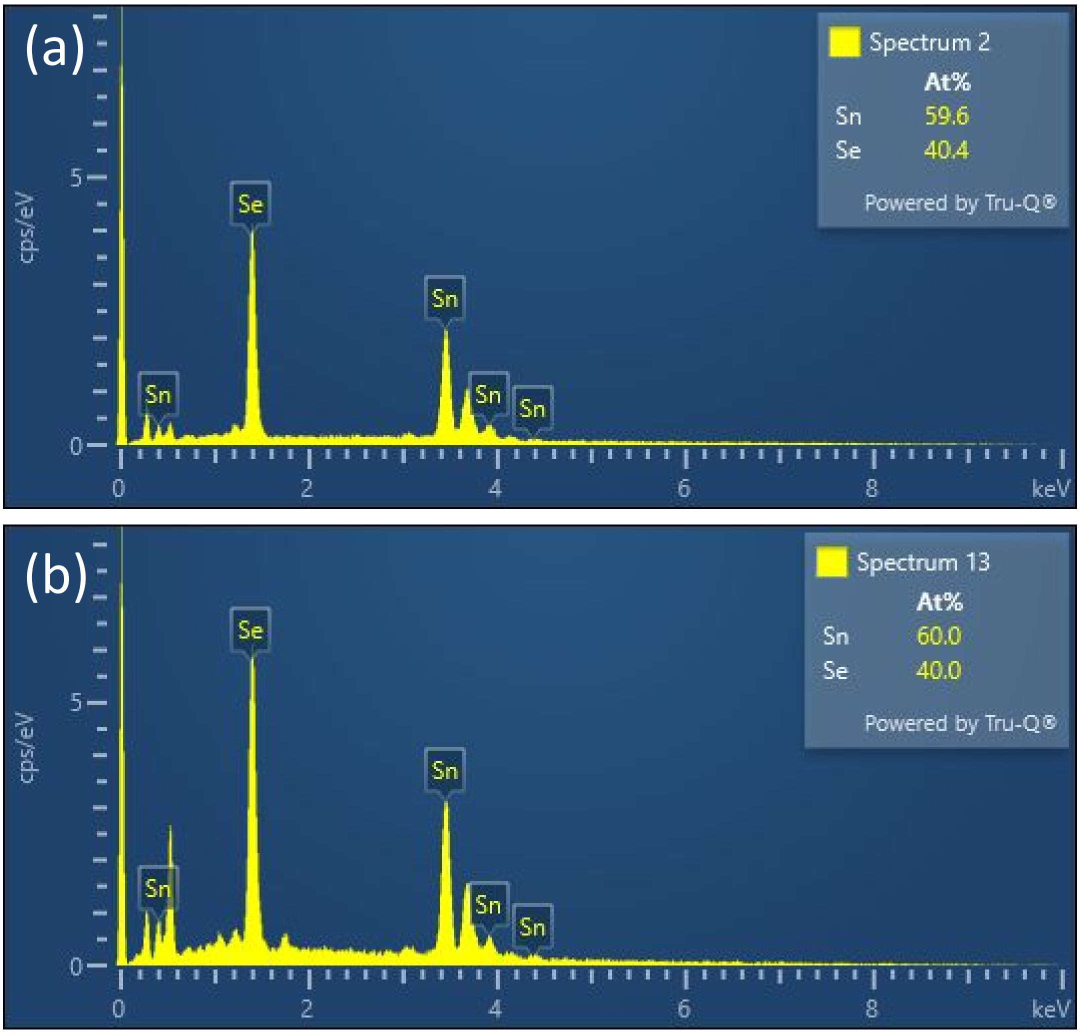

Figure S3. Energy Dispersive X-Ray Spectroscopy of a) ball milled Sn and Se powder (Figure S3d), and b) commercially sourced SnSe powder (Sigma-Aldrich, 99.995%).

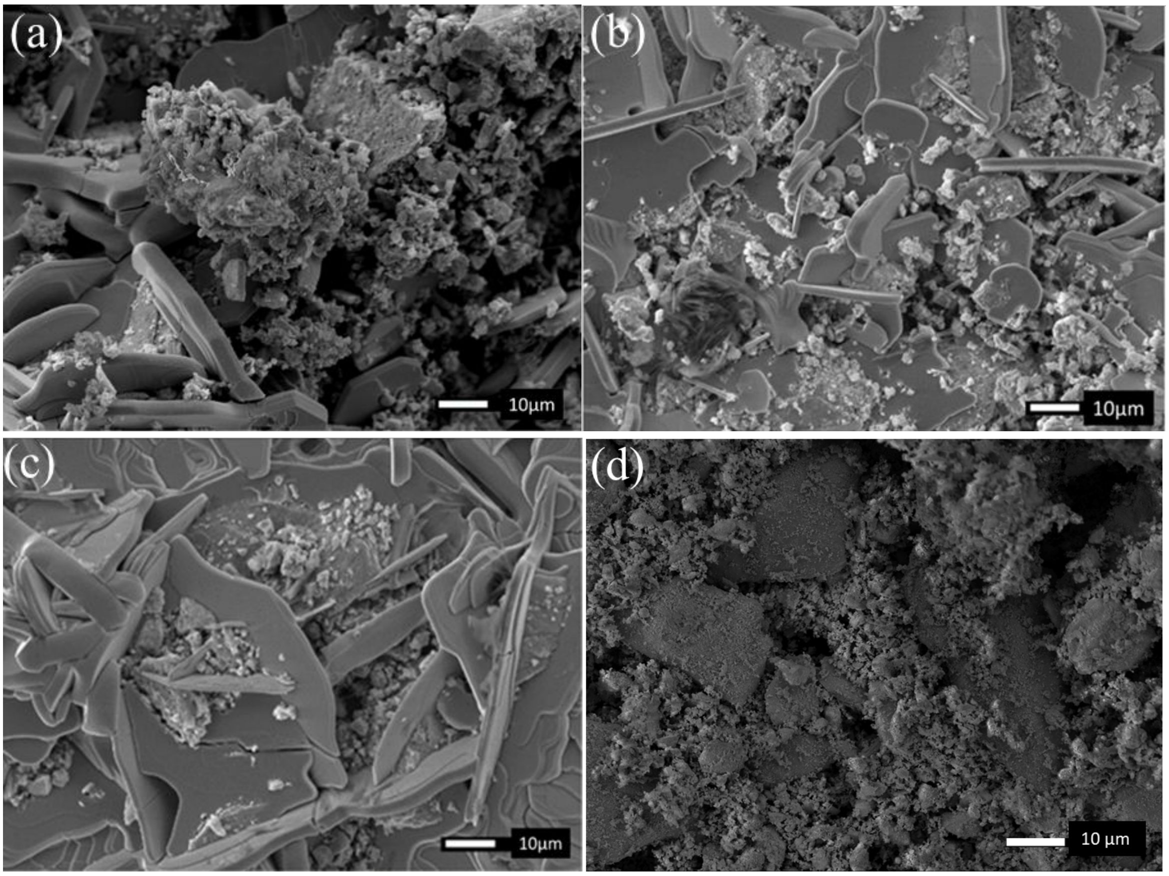

Figure S4. SEM, a-c) of printed cured SnSe samples with Na2SiO3 binder percentages of a) 2.0%, b) 3.0% and c) 4.0% (other binder percentage can be seen in Figure 2), d) SEM of ball milled powder.

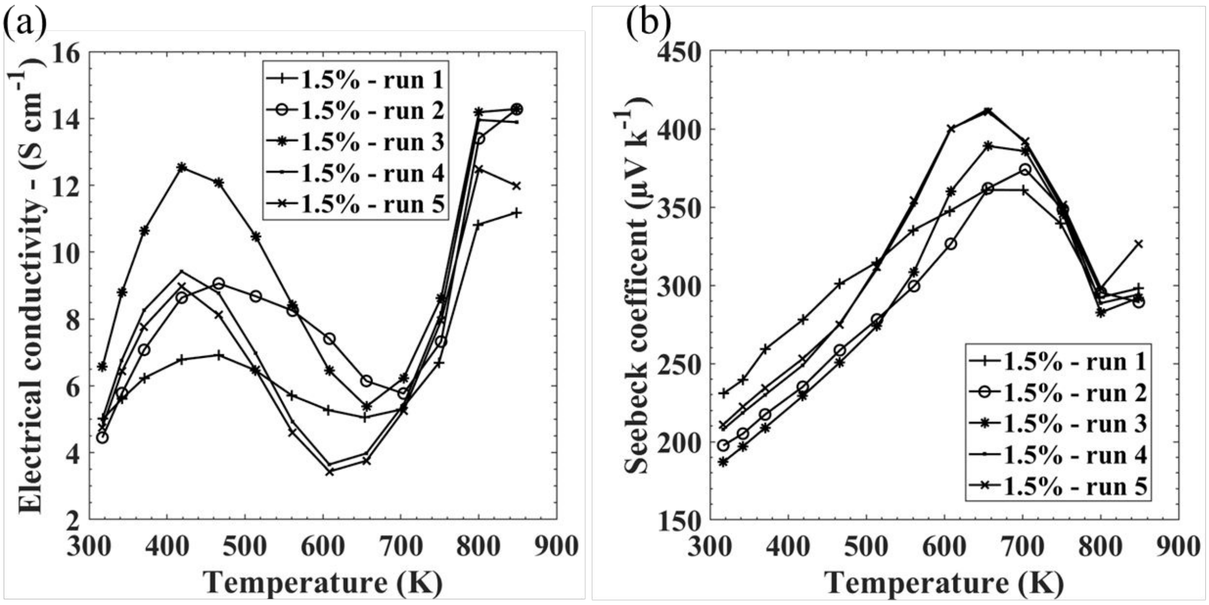

Figure S5. Thermoelectric performance over several thermal cycles of pseudo 3D printed SnSe with 1.5% Na2SiO3 binder: a) electrical conductivity and b) Seebeck coefficient.

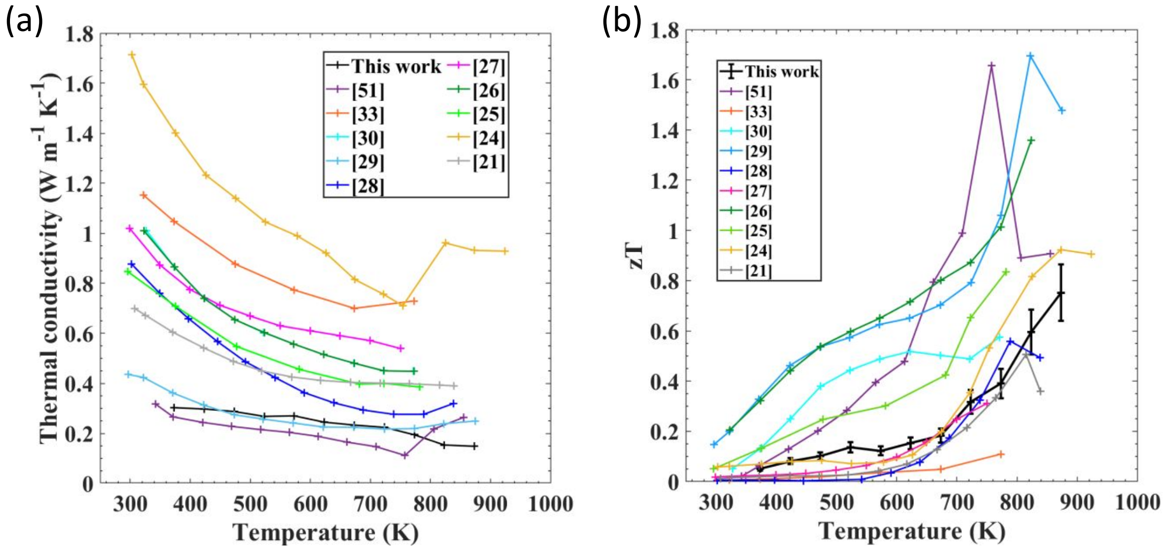

Figure S6. (a) Thermal conductivity and (b) figure of merit performanace of rapid psuedo-3D printed SnSe compared to other polycrystaline SnSe reported in the literature. Error bars in (b) represent the ±15% uncertainty of the measurement.

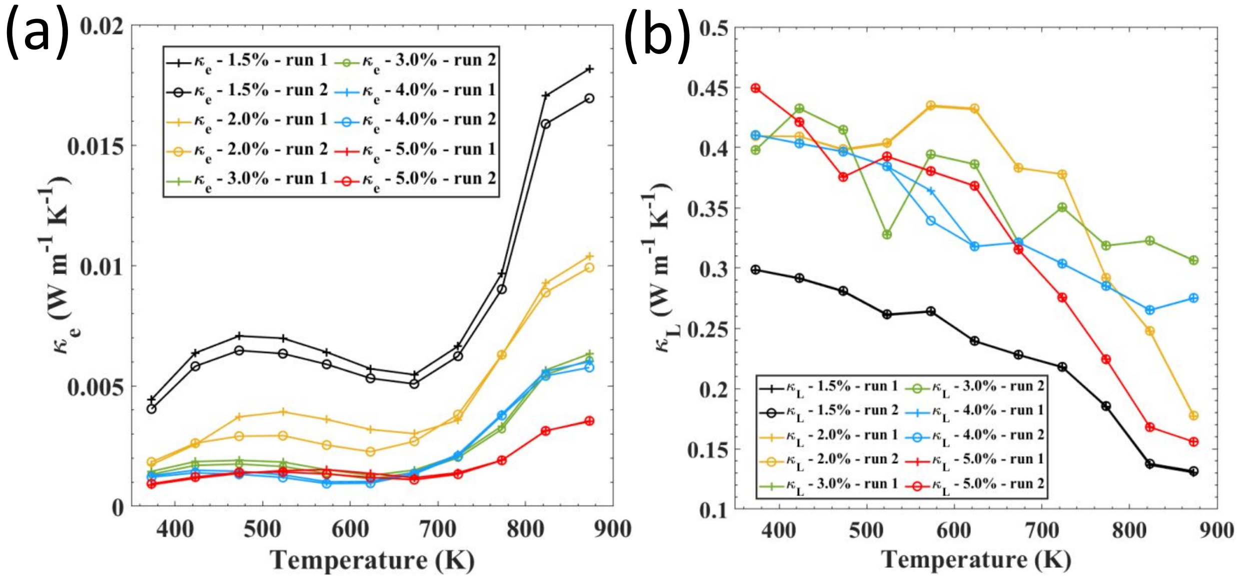

Figure S7. Thermal conducutivity components of pseudo-3D printed SnSe using a Na2SiO3 binder (cross markers represent the first runs, whilst the circular markers represent the second runs): a) κe (electronic component), b) κL (lattice component).

Table S1. Table of densities used for calculation of thermal conductivity.

Binder concentration	Density
1.50%	5.329
2.00%	5.259
3.00%	5.312
4.00%	5.263
5.00%	5.232

Table S2. SnSe thermoelectric generator (TEG) characterization: hot side temperature (TH), cold side temperature (TC), open circuit voltage (VOC) output of TEG, short circuit current (ISC) output of TEG and peak power outputs of the TEG (assuming maximum power = ISCVOC/4).56

T_H (K)	T_C (K)	Voltage (mV)	Current (μA)	power (μW)
294.15	294.15	0.8	0.2	0.00004
319.15	298.15	18.8	8.4	0.03948
364.15	307.15	55.4	29.7	0.411345
417.15	318.15	95.2	65.9	1.56842
458.15	329.15	132	64.8	2.1384
498.15	335.15	148.32	88.3	3.274164
548.15	342.15	245.2	760	46.588
604.15	348.15	244.8	1000	61.2
646.15	360.15	141.2	3010	106.253
700.15	384.15	175.2	3021	132.3198
736.15	405.15	248.2	4013	249.00665
789.15	421.15	264.2	7065	466.64325
